# Factors Affecting Women’s Assessment and Satisfaction with Their Childbirth

**DOI:** 10.3390/medicina60010086

**Published:** 2024-01-01

**Authors:** Jagoda Konieczka, Katarzyna Tomczyk, Maciej Wilczak, Karolina Chmaj-Wierzchowska

**Affiliations:** Department of Maternal and Child Health, Poznan University of Medical Sciences, 61-701 Poznan, Poland

**Keywords:** childbirth, satisfaction, delivery

## Abstract

*Background and Objective:* Childbirth is one of the most significant experiences in a woman’s life. The manner in which childbirth unfolds and is experienced can be influenced by various factors, including the birthing environment and the woman’s attitude and preparation. Taking a holistic view of childbirth, it becomes apparent that addressing the basic physiological needs during childbirth can significantly influence the comfort and sense of security of laboring women. The aim of this research was to assess the level of satisfaction among women with their experience during childbirth and to identify its determinants. *Materials and Methods:* This study included 275 women who had given birth within the past 15 years and were up to 40 years of age. The research method employed was a diagnostic survey, involving a self-designed questionnaire. *Results:* discussing the birth plan with the midwife, the ability to ask questions during labor, consuming meals during labor, water immersion, listening to music during labor, assuming vertical positions during the second stage of labor, and skin-to-skin contact are associated with increased satisfaction with the childbirth experience. *Conclusions:* The study findings revealed that the highest levels of satisfaction were reported in connection with the interactions with medical staff during childbirth and the quality of facilities available during delivery. Conversely, the lowest levels of satisfaction were associated with the possibility of using pain relief methods during labor.

## 1. Introduction

Physiological labor is defined as the spontaneous onset of uterine contractions and the delivery of a baby between 37 and 42 weeks of gestation, typically associated with minimal risks. A successful normal labor ends with the natural birth of the baby, with both the mother and baby in good health [1]. The progression of labor, in addition to normal uterine contractions and appropriate cervical dilation, is influenced by a variety of factors, including the pelvic structure of the parturient, the fetal position and alignment, the attitude of the parturient, the support received from the attendant, and the effective teamwork of the medical staff, among other considerations [2,3]. Pain is an inherent aspect of the labor process, and its intensity and sensation are subjective and influenced by psychological factors. The level of pain experienced may depend on the attitude of the parturient, the support of a loved one, her overall life circumstances, her emotional state, the birthing environment, as well as the pain threshold of the patient [4]. 

Childbirth is one of the most important events biologically in a woman’s life. It is a transformative moment in which a woman becomes a mother. This emotional experience not only shapes and affects the woman’s future but also impacts her newborn child. The course of childbirth also largely depends on the parturient and her level of preparation. Nowadays, more and more attention is being paid to building awareness among women through prenatal education. The experience of childbirth in an environment that promotes comfort and safety helps build strong and confident women and mothers. In contrast, childbirth accompanied by anxiety, fear, and unexpected complications can lead to depression, diminished mood, and lowered self-esteem, making it difficult to feel happiness and satisfaction with the birth of a child. Some women describe their childbirth as the most beautiful event, while others describe it as a traumatic experience. The factors that may affect a woman’s satisfaction with her childbirth experience include her ability to eat during labor; the availability of pharmacological and nonpharmacological pain relief methods, including breathing techniques [4,5,6,7,8], water immersion [8,9,10,11], music therapy [12,13,14], aromatherapy [15,16,17,18], acupuncture and acupressure [8,9,19], transcutaneous electrical nerve stimulation (TENS) [9,20,21], and the option for assuming vertical positions during the first and second stages of labor [8,22,23,24]; skin-to-skin contact between the newborn and the mother; and the opportunity to ask questions during labor and to discuss the birth plan and expectations concerning labor. In Poland, in accordance with the regulations set forth by the Minister of Health regarding the organizational standard of perinatal care, we proposed the preparation of a birth plan that includes the parturient’s preferences and expectations, as well as all aspects of medical management during labor and the choice of delivery location [25].

The aim of this study was to assess the level of satisfaction of women with their childbirth experiences and to identify the key determinants thereof.

## 2. Materials and Methods

This study included 275 women who had undergone various modes of childbirth such as physiological and surgical vaginal deliveries, as well as emergency cesarean section deliveries. The survey was administered using Google Forms, ensuring anonymous and voluntary participation, with women being randomly selected for this study. Women who had undergone elective cesarean sections were not included in the research. The inclusion criteria consisted of women under the age of 40, with no more than 15 years having elapsed since their last delivery. All respondents were Polish women with public insurance. The respondents gave birth in public hospitals where they were provided with the care of a midwife and an obstetrician–gynecologist.

The self-report survey consisted of 37 questions. The initial questions focused on collecting sociodemographic data from the participants (age, marital status, place of residence, education, age at the time of delivery, and the presence of a companion during delivery). Subsequent questions focused on childbirth-related factors, encompassing aspects such as the number of previous childbirths, and the type and progression of labor. The most extensive section of the survey pertained to issues of labor comfort, covering topics like childbirth preparedness, medical interventions, the availability of pharmacological and nonpharmacological pain relief methods, and opportunities for skin-to-skin contact. The overall level of women’s satisfaction with childbirth experience was assessed concerning their birth plan, interactions with medical personnel, and the quality of amenities in the hospital or birthing room.

Data analysis was conducted using TIBCO Software Inc. (2017) Statistica, version 13, and Microsoft Excel, version 2019 (Microsoft Office). For group comparisons, the Mann–Whitney U test and Kruskal–Wallis ANOVA test were used. A chi-square test, recognized for its high reliability, was used to assess the relationships between variables. The significance level for all calculations was set at *p* < 0.05.

## 3. Results 

### 3.1. Characteristics of the Study Group

This study included 275 postpartum women with different diverse educational backgrounds, marital statuses, and places of residence. The characteristics of the study group are shown in Table 1.

### 3.2. Factors Affecting Satisfaction with the Childbirth Experience

In the evaluation of factors that could influence satisfaction with childbirth, we distinguished the following key determinants: the ability to eat during labor, water immersion, listening to music, aromatherapy, acupuncture, TENS, the use of painkillers, assuming vertical positions (sitting or standing) during the first and second stages of labor, skin-to-skin contact, the opportunity to ask questions during labor, and discussions about the birth plan with the midwife. The applicability of these factors as reported by the respondents is shown in Table 2.

### 3.3. Satisfaction with the Delivery According to Sociodemographic Data and Type of Delivery and Perinatal Activities

Respondents assessed their satisfaction with their childbirth experience on a 10-point scale, where 0 signified very dissatisfied and 10 indicated very satisfied. Their responses were categorized into the following areas: 

I. Satisfaction with the ability to use pain relief methods in the course of childbirth;

II. Satisfaction in relation to the alignment of expectations and the birth plan with the actual course of labor; 

III. Satisfaction with interactions with medical personnel during childbirth; 

IV. Satisfaction with available facilities during childbirth;

V. Overall satisfaction with the childbirth experience.

The study results pertaining to the influence of sociodemographic data and the type of delivery on satisfaction with the delivery are shown in Table 3. 

It was observed in this study that respondents with higher levels of education displayed a significantly higher satisfaction with childbirth, particularly in terms of the alignment of expectations with the birth plan. This study observed a significant relationship between place of residence and satisfaction, with women residing in rural areas expressing a significantly higher satisfaction with the available amenities during childbirth (*p* = 0.01) and overall satisfaction with the childbirth experience (*p* = 0.043) in comparison to those living in cities with populations of up to 150,000. This study found no statistically significant relationship between marital status and satisfaction with childbirth. It was also observed that multiparous women reported significantly higher levels of satisfaction in various aspects of childbirth, such as the ability to use pain relief methods, the alignment of expectations with the birth plan, interactions with medical personnel, and overall satisfaction with the childbirth experience. Furthermore, respondents who gave birth in university hospitals displayed a significantly higher satisfaction with childbirth in the aspect of alignment of expectations with the birth plan as well as with the available amenities during childbirth. The presence of a companion during childbirth significantly increased the satisfaction with the possibility of using available amenities during childbirth (*p* = 0.02). Women who experienced natural childbirth exhibited significantly higher satisfaction levels across various aspects, including the ability to use pain relief methods, the alignment of expectations with the birth plan, interactions with medical personnel, and overall satisfaction with the childbirth experience. However, women who underwent perineal incisions reported a significantly lower satisfaction with the possibility of using pain relief methods during labor compared to those who experienced labor with perineal protection (*p* < 0.001) and perineal rupture (*p* < 0.001). 

Subsequently, this study examined the effects of individual perinatal activities on patients’ perceived satisfaction with childbirth. The results are shown in Table 4.

This study demonstrated that respondents who used painkillers expressed a significantly lower overall satisfaction with their childbirth experience, although the use of painkillers did not affect other components of satisfaction. This study demonstrated a significant relationship between discussions of the birth plan with the midwife and satisfaction with the delivery process. Women who did not discuss the birth plan reported a significantly lower satisfaction in multiple aspects, including the possibility of using pain relief methods, the alignment of expectations with the birth plan, interactions with medical staff, available facilities, and overall satisfaction with the childbirth experience (*p* < 0.001). Moreover, women who had the opportunity to ask questions during labor displayed significantly higher satisfaction levels across various aspects such as the possibility of using pain relief methods, the alignment of expectations with the birth plan, interactions with medical staff, the available amenities, and overall satisfaction with the childbirth experience (*p* < 0.001). 

Women who were able to consume meals during labor expressed significantly higher satisfaction levels concerning the ability to use pain relief methods, the alignment of expectations with the birth plan, and interactions with medical personnel compared to women who could not eat (*p* < 0.001). Satisfaction with the available amenities during labor (*p* < 0.01) and overall satisfaction with labor (*p* < 0.03) were also statistically significantly higher for these participants. 

This study found that the use of water immersion and listening to music during labor were associated with significantly higher levels of satisfaction across multiple aspects, including the possibility of using pain relief methods, the alignment of expectations with the birth plan, interactions with medical personnel, possible use of the available facilities, and overall satisfaction with childbirth (*p* < 0.001). In contrast, respondents who used painkillers reported a significantly lower overall satisfaction with the childbirth experience (*p* < 0.001). Additionally, this study demonstrated that respondents who adopted vertical positions during the first stage of labor reported significantly higher satisfaction levels with regard to the alignment of expectations with the birth plan, use of the available amenities (*p* < 0.01), interactions with medical personnel (*p* < 0.001), and overall satisfaction with the labor experience (*p* < 0.02). 

This study demonstrated that respondents who adopted vertical positions during the second stage of labor exhibited significantly higher satisfaction levels with respect to the ability to use pain relief methods (*p* < 0.01), the alignment of expectations with the birth plan, interactions with medical personnel, the use of available amenities, as well as an overall higher satisfaction with childbirth (*p* < 0.001). 

This study also observed that respondents who had the opportunity for skin-to-skin contact with their newborns expressed significantly higher satisfaction levels across various aspects such as the ability to use pain relief methods, the alignment of expectations with the birth plan, having interactions with medical personnel, the use of available facilities, as well as overall satisfaction with the childbirth experience (*p* < 0.00, *p* = 0.001). 

In summary, the following perinatal factors had a statistically significant impact on all aspects of satisfaction with the birth that we studied: discussing the birth plan with the midwife, the ability to ask questions during labor, consuming meals during labor, water immersion, listening to music during labor, assuming vertical positions during the second stage of labor, and skin-to-skin contact. 

## 4. Discussion

Childbirth is one of the most important experiences and events biologically in a woman’s life, and the way it is remembered is influenced by numerous factors, many of which are addressed to varying degrees in perinatal care. Currently, there is a growing discussion about the need to raise medical standards, which mean the performance of perinatal procedures and facilities during childbirth among healthcare professionals, as well as to enhance awareness among laboring women. This emphasis on improving the childbirth experience is especially important as it directly impacts the quality of life for both women and newborns following delivery.

In this study, the term “satisfaction with completed labor” is understood as satisfaction with the possibility of using the pain relief methods during labor, the alignment of expectations with the birth plan with the actual labor experience, the contentment with interactions with medical personnel, the utilization of perinatal facilities, and overall satisfaction with the childbirth process. When sociodemographic factors were examined, age and marital status were not found to affect satisfaction with the delivery. However, a study conducted by Wszołek found that married women were more likely to feel joy (94%) and happiness (90.3%) about undergoing childbirth than were unmarried (89.1%, and 78.3%) and divorcees (81.3%, and 81.3%) [26].

By analyzing other sociodemographic factors, the relationship between education and satisfaction with childbirth was tested and it was found that women with higher education levels reported greater satisfaction with their childbirth experience. Similar conclusions were reached by Kraśnianin et al., who compared the satisfaction of Polish and German women with perinatal care. Their study showed that higher-educated Polish women (47%), and German women (26%) showed a higher degree of confidence in the medical staff and the care provided. Among them, 91% of Polish and 97% of German women rated the care positively and expressed high levels of satisfaction [27]. 

The presence of a companion during labor was also identified as a factor that increases the sense of satisfaction with the delivery. Similar findings were also obtained by Dubey et al. [28]. The impact of a husband’s presence during childbirth was studied by Sapkota et al., who used the LAS scale (Labour Agentry Scale) to measure the degree of control women felt during labor. This study included primiparous women who gave birth in the presence of their husband (*n* = 97), in the presence of a friend (*n* = 96), with mixed support (*n* = 11), and a control group (*n* = 105). In the study conducted, a higher level of control over labor was displayed by women who gave birth in the presence of their husband (*β* = 0.54; *p* < 0.001) than those who were with a friend and these women were associated with a lower feeling of control during labor (*p* < 0.001). Women who give birth with their husbands are characterized by greater resourcefulness and control during childbirth, which affects their sense of satisfaction and overall satisfaction with childbirth [29]. Similar results were obtained in a diagnostic survey by Król et al. in a group of 91 pregnant women. In their study, as many as 93.41% of women were aware of the possibility of giving birth with a companion. They considered the presence of a companion during childbirth as a particularly important aspect that helps alleviate stress, reduce suffering, and improve the overall birthing experience, ultimately facilitating delivery [30].

The method of completing labor also has an impact on a woman’s satisfaction with her childbirth experience, with natural childbirth often yielding more favorable results compared to surgical deliveries. An interesting finding was presented by Bossano et al. who studied satisfaction levels in women a decade after delivery. The study group consisted of 576 women. It was found that those who had given birth vaginally reported a significantly higher fulfillment (0.40 vs. 0.15, *p* < 0.001) and less stress (−0.34 vs. 0.20, *p* < 0.001) than those who had undergone cesarean section deliveries. This finding underscores the enduring influence of the method of birth completion on women’s satisfaction and enjoyment even several years after childbirth [31].

In the current study, a distinct association was observed between the course of childbirth and the level of satisfaction. Specifically, women who had undergone a perineal incision procedure expressed a significantly lower satisfaction with their delivery compared to women who had experienced perineal protection or spontaneous rupture. Similar results were obtained by Gutt, who studied the effect of perineal incisions on women’s physical and psychosexual well-being. In the results, as many as 52.5% of women reported feelings of mutilation, 37.5% reported a violation of intimacy, and 35% complained of the occurrence of sexual problems following perineal incisions [32]. The results of perineal incision on women’s quality of life after childbirth were examined by Chang et al. who studied the incidence of sexual dysfunction after childbirth and the occurrence of urinary incontinence in a group of 243 women. The results indicated that perineal incisions resulted in increased pain at 1, 2, and 6 weeks after delivery and urinary incontinence at 3 months post delivery. These physical discomforts significantly reduced satisfaction with the childbirth experience and overall postpartum satisfaction [33]. Similar findings were reported by Fumagalli et al., who studied women’s satisfaction with their childbirth experience in relation to the quality of care and the stress experienced during childbirth. The results indicated that women with an intact perineum were more satisfied with their childbirth (*p* = 0.008) [34]. This study clearly shows the detrimental effect of perineal incisions on women’s postpartum quality of life, highlighting the importance of avoiding routine perineal incisions and instead opting for perineal protection to enhance women’s satisfaction. 

The ability for women to freely assume positions during the first and second stages of labor also emerges as an important factor in influencing satisfaction with childbirth. A study by Liu et al. revealed that the free adoption of vertical positions in the first stage of labor was possible by 84.3% of the subjects and by 67.9% in the second stage. It was reported that the ability to assume comfortable positions had a significant effect on increasing satisfaction with childbirth among women [35]. Similar conclusions were drawn by Nieuwenhuijze et al., who studied the comfort level of women who adopted relaxed positions during the second stage of labor [36].

Water immersion is associated with a positive impact on perceived satisfaction with childbirth. Antonakou et al. conducted a study involving a group of 12 Greek women who utilized water immersion during labor, with 9 of them giving birth in water. All the women described their labor as a wonderful experience during which they felt in control and played an active role. Even women who did not ultimately give birth in water but used water immersion during labor reported high levels of satisfaction with their birth [37]. Dado et al. also noted the positive effects of water immersion in their study [38].

An interesting finding in this study is the effect of the use of painkillers. Patients who used painkillers had a significantly lower overall satisfaction with labor. It is possible that this is related to the choice of the drug or the dose being inappropriate for a given patient. In a study by Rogala et al., painkillers were used by more than half of the women, but the frequency of painkiller use was not associated with an increased sense of satisfaction with the delivery [39]. Madden et al. studied the preferences of women, midwives, and obstetricians regarding pain relief methods, with pregnant women comprising 31% of the study group. The survey found that the use of pharmacological methods of pain relief (pethidine/morphine) was the least preferred, and women tended to value nonpharmacological methods of pain relief more [40]. 

This study also found that patients who prepared for labor during pregnancy were characterized by a lower overall satisfaction with childbirth. This might be related to the increased awareness among these women and the difficulty in implementing an ideal birth plan. Different results were obtained by Duncan et al., who compared childbirth preparation through specialized courses versus routine preparation. Women who attended the workshop reported a greater satisfaction with childbirth and better mental states after delivery than those in the control group. The preparation for childbirth contributed to an increased efficiency and awareness during childbirth. In addition, they had a lower risk of postpartum depression and they were also less likely to use painkillers [41]. A similar study was conducted by Munkhondya et al. compared to the sense of fear of childbirth, the sense of efficacy in childbirth, and the support of a companion or a partner in specialized preparations for childbirth versus standard perinatal care. Although no significant differences were noted in the course of labor, patients who engaged in an additional childbirth preparation reported a higher sense of efficacy in childbirth and reduced anxiety [42]. Wszołek also noted a positive correlation between childbirth preparation and feelings of calmness, confidence, and satisfaction with childbirth [26].

Having and discussing a birth plan with a midwife also had a positive impact on satisfaction with childbirth. It appears that women who did not discuss the birth plan with the midwife were characterized by a lower satisfaction than parturients who did discuss it. Sioma-Markowska et al. highlighted in their study that 60% of midwives did not inquire about the parturient’s birth plan, and in 81% of cases, expectations about the birthing process were not discussed. This lack of communication left women dissatisfied with the implementation of the procedure, which resulted in reduced satisfaction [43]. Alba-Rodríguez et al. studied women’s experiences with birth plans and described them as valuable communication tools between the parturient and the midwife through which women can state their wishes regarding the birth. With these discussions about the plan, patients felt calmer and they had a more positive perception of the birthing experience as well as better interactions with the medical staff [44].

Women who had the opportunity for “skin-to-skin” contact showed a higher level of satisfaction with their delivery. In a study by Mazúchová et al. investigating satisfaction with psychosocial aspects of care during childbirth in a group of 360 women, it was observed that “skin-to-skin” contact was not promoted in 52.2% of women, causing these women to perceive their childbirth as complicated. On the other hand, patients who had the opportunity for immediate skin-to-skin contact after delivery reported higher levels of satisfaction with their delivery experience [45]. Kahalon et al. provided further evidence of the importance of skin-to-skin contact in achieving a positive childbirth experience. They studied the relationship between skin-to-skin contact and satisfaction from the type of delivery. Among 1371 women, the frequency of using skin-to-skin contact after a physiological delivery was 83%, that after a surgical delivery was 66%, and that after a cesarean section was 31%. It was found that in each group, skin-to-skin contact increased satisfaction with childbirth [46].

A very important aspect of life is mental health, particularly during the perinatal period when women are vulnerable to experiencing a deterioration in their overall well-being due to the many changes that occur during this time. Therefore, an important aspect is to improve the quality of perinatal care for patients to increase the level of their satisfaction with childbirth, which will undoubtedly have a positive influence on their quality of life. 

## 5. Conclusions

Discussing the birth plan with the midwife, the ability to ask questions during labor, consuming meals during labor, water immersion, listening to music during labor, assuming vertical positions during the second stage of labor, and skin-to-skin contact are associated with increased satisfaction with the childbirth experience. 

The study findings revealed that the highest levels of satisfaction were reported in connection with the interactions with medical staff during childbirth and the quality of facilities available during delivery. Conversely, the lowest levels of satisfaction were associated with the possibility of using pain relief methods during labor.

## Figures and Tables

**Table 1:** Characteristics of the study group.

Age	Age at the Time of the Survey	30.4 ± 4.29 M ± SD	Age at the Time of Labor	28.41 ± 4.16 M ± SD
Education	Higher 66.55%	Secondary 29.09%	Vocational 4.36%	
Place of residence	Village 33.45%	City <50 thousand inhabitants 15.64%	City 50–500 thousand inhabitants 29.82%	City >500 thousand inhabitants 21.09%
Marital status	Married 78%	Informal relationship 18.55%	Single 4%	Divorced 1.4%
Childbearing	>3 1.09%	3 8%	2 29.82%	1 61.09%
Child’s place of birth	University Hospital 42.91%	Nonuniversity hospital 45.46%	Home 1.45%	Other 1.09%
Presence of an accompanying person	Partner/Husband 76.36%	Other accompanying person 2.55%	No accompanying person 21.09%	
Kind of birth	Natural childbirth77.45%	Surgical vaginal delivery—Vacuum Extractor 6.55%	Surgical vaginal delivery—Forceps 2.55%	Emergency cesarean section 13.45%
Course of physiological childbirth	With perineal incision 35.27%	With perineal protection 25.45%	With perineal rupture 18.55%	

**Table 2:** Frequency of use of selected perinatal activities by respondents.

Perinatal Activities	Yes	No	No Possibility
Eating a meal during labor	69.09%	17.09%	13.82%
Water immersion	38.55%	45.45%	16%
Listening to music	33.45%	39.28%	27.27%
Aromatherapy	4%	45,01%	50.91%
Acupuncture	2.18%	97.82%	53.45%
TENS	8.36%	44%	47.64%
Painkillers	64.73%	35.27%	-
Vertical position in the first stage of labor	86.55%	13.45%	-
Vertical position in the second stage of labor	69.82%	30.18%	-
Skin-to-skin contact	82.91%	17.09%	-
Opportunity to ask questions during labor	80%	20%	-
Discussing the birth plan with the midwife	43.27%	56.73%	-

**Table 3:** Satisfaction with the delivery according to sociodemographic data and type of delivery.

		I M ± SD	II M ± SD	III M ± SD	IV M ± SD	V M ± SD
Age at the time of labor	<25 years	5.77 ± 3.42	5.89 ± 3.59	6.82 ± 3.26	7.47 ± 2.99	6.95 ± 3.18
	26–29 years	5.73 ± 3.25	6.14 ± 3.35	7.28 ± 3.28	7.64 ± 2.96	6.93 ± 3.16
	>30 years	5.98 ± 3.01	6.51 ± 2.9	7.69 ± 2.63	7.89 ± 2.31	7.72 ± 2.45
	*p*-value	0.92	0.73	0.37	0.93	0.38
Education	Higher	5.95 ± 3.1	6.51 ± 3.12	7.46 ± 2.96	7.78 ± 2.59	7.43 ± 2.71
	Other	5.61 ± 3.39	5.64 ± 3.42	7.04 ± 3.22	7.53 ± 3.02	6.85 ± 3.31
	*p*-value	0.51	0.049	0.26	0.91	0.45
Place of residence	Village	6.22 ± 3.19	6.66 ± 3.3	7.78 ± 2.81	8.09 ± 2.59	7.68 ± 2.83
	City 150 thousand	5.13 ± 3.24	5.68 ± 3.07	6.91 ± 2.97	6.95 ± 2.89	6.82 ± 2.89
	City > 150 thousand	6.09 ± 3.09	6.28 ± 3.31	7.26 ± 3.3	7.97 ± 2.63	7.17 ± 3.04
	*p*-value	0.045	0.07	0.08	0.01	0.045
Marital status	Formal relationship	5.93 ± 3.21	6.44 ± 3.12	7.44 ± 2.95	7.73 ± 2.69	7.47 ± 2.75
	Informal relationship	5.55 ± 3.15	5.52 ± 3.54	6.95 ± 3.35	7.59 ± 2.9	6.5 ± 3.36
	*p*-value	0.34	0.07	0.62	0.95	0.07
Childbearing	Primipara	5.38 ± 3.15	5.57 ± 3.39	6.65 ± 3.3 8	7.48 ± 2.83	6.65 ± 3.16
	Multipara	6.55 ± 3.15	7.25 ± 2.72	8.37 ± 2.26	8.03 ± 2.56	8.15 ± 2.26
	*p*-value	0.002	<0.001	<0.001	0.09	<0.001
Child’s place of birth	University hospital	6.26 ± 2.73	6.74 ± 3.08	7.49 ± 3.06	8.25 ± 2.28	7.53 ± 2.72
	Other hospital	5.37 ± 3.41	5.72 ± 3.27	7.15 ± 3.06	7.17 ± 3	6.95 ± 3.04
	*p*-value	0.054	0.01	0.26	0.01	0.15
Presence of an accompanying person	Yes	5.91 ± 3.12	6.38 ± 3.16	7.36 ± 3.04	7.94 ± 2.52	7.23 ± 2.9
	No	5.55 ± 3.46	5.64 ± 3.51	7.19 ± 3.14	6.78 ± 3.29	7.26 ± 3.08
	*p*-value	0.57	0.15	0.63	0.02	0.73
Kind of birth	Natural childbirth	6.24 ± 3.11	6.9 ± 2.95	7.8 ± 2.75	7.84 ± 2.66	7.77 ± 2.57
	Surgical: surgical vaginal delivery or cesarian section	4.44 ± 3.11	3.89 ± 3.17	5.68 ± 3.47	7.21 ± 2.97	5.4 ± 3.36
	*p*-value	<0.001	<0.001	<0.001	0.16	<0.001
Course of physiological childbirth	Perineal protection	7.14 ± 2.92	8.39 ± 2.25	8.9 ± 1.78	8.63 ± 2.13	8.9 ± 1.79
	Perineal incision	5.02 ± 2.94	5.18 ± 3.04	6.37 ± 3.07	7.07 ± 2.84	6.23 ± 3.02
	Perineal rupture	6.82 ± 3.15	7.33 ± 2.73	8.63 ± 2.37	8.55 ± 2.12	8.35 ± 2.09
	*p*-value	<0.001	<0.001	<0.001	<0.001	<0.001

I. Satisfaction with the ability to use pain relief methods in the course of childbirth. II. Satisfaction in having expectations and the birth plan well aligned to the actual course of labor. III. Satisfaction from interactions with medical personnel during childbirth. IV. Satisfaction from the possibility of using facilities during childbirth. V. Satisfaction with the overall birth experience. M—median, SD—standard deviation.

**Table 4:** Dependence of satisfaction with birth on perinatal activities.

		I M ± SD	II M ± SD	III M ± SD	IV M ± SD	V M ± SD
Discussing the birth plan with the midwife	yes	6.58 ± 2.93	7.24 ± 2.78	8.13 ± 2.44	8.3 ± 2.36	7.87 ± 2.45
	no	4.35 ± 3.19	4.3 ± 3.14	5.59 ± 3.44	6.16 ± 3.18	5.72 ± 3.19
	no birth plan	6.21 ± 3.12	6.62 ± 3.21	7.86 ± 2.79	8.32 ± 2.13	7.81 ± 2.81
	*p*-value	<0.001	<0.001	<0.001	<0.001	<0.001
Asking questions during labor	yes	6.67 ± 2.78	7.14 ± 2.79	8.26 ± 2.21	8.27 ± 2.18	8.01 ± 2.25
	no	2.49 ± 2.47	2.56 ± 2.19	3.56 ± 3.07	5.4 ± 3.47	4.13 ± 3.27
	*p*-value	<0.001	<0.001	<0.001	<0.001	<0.001
Eating a meal during labor	yes	6.46 ± 2.94	6.97 ± 2.93	7.98 ± 2.47	8.62 ± 2.01	7.87 ± 2.23
	no	3.66 ± 3.05	3.92 ± 3.03	5.26 ± 3.53	6.24 ± 3.54	5.26 ± 3.64
	*p*-value	<0.001	<0.001	<0.001	0.01	0.003
Water immersion	yes	6.07 ± 3	6.4 ± 3.22	7.32 ± 3.09	8.45 ± 2.05	6.95 ± 3.16
	no	4.16 ± 3.12	4.29 ± 2.99	5.74 ± 3.26	5.97 ± 3.2	6.09 ± 3.03
	*p*-value	<0.001	<0.001	<0.001	<0.001	<0.001
Listening to music during labor	yes	6.42 ± 2.77	6.75 ± 2.97	7.65 ± 2.76	8.83 ± 1.65	7.62 ± 2.63
	no	4.36 ± 3.22	4.53 ± 3.15	5.92 ± 3.3	6.1 ± 3.25	5.92 ± 3.25
	*p*-value	<0.001	<0.001	<0.001	<0.001	<0.001
Painkillers	yes	5.87 ± 2.95	5.97 ± 3.24	7.22 ± 3.03	7.79 ± 2.61	6.92 ± 3.03
	no	5.77 ± 3.61	6.69 ± 3.22	7.51 ± 3.1	7.52 ± 2.97	7.82 ± 2.66
	*p*-value	0.83	0.06	0.30	0.70	0.01
Vertical position in the first stage of labor	yes	5.97 ± 3.2	6.45 ± 3.17	7.6 ± 2.86	7.89 ± 2.6	7.44 ± 2.78
	no	5 ± 3.11	4.78 ± 3.41	5.54 ± 3.63	6.43 ± 3.25	5.95 ± 3.54
	*p*-value	0.07	0.01	0.001	0.01	0.02
Vertical position in the second stage of labor	yes	6.15 ± 3.2	6.69 ± 3.14	7.82 ± 2.73	8.12 ± 2.43	7.71 ± 2.66
	no	5.12 ± 3.08	5.14 ± 3.26	6.18 ± 3.44	6.71 ± 3.14	6.14 ± 3.25
	*p*-value	0.01	<0.001	<0.001	<0.001	<0.001
Skin-to-skin contact	yes	6.15 ± 3.09	6.63 ± 3.04	7.75 ± 2.74	8 ± 2.47	7.59 ± 2.64
	no	4.32 ± 3.31	4.26 ± 3.54	5.23 ± 3.6	6.21 ± 3.43	5.53 ± 3.62
	*p*-value	0.001	<0.001	<0.001	0.001	<0.001
Painkillers	yes	5.87 ± 2.95	5.97 ± 3.24	7.22 ± 3.03	7.79 ± 2.61	6.92 ± 3.03
	no	5.77 ± 3.61	6.69 ± 3.22	7.51 ± 3.1	7.52 ± 2.97	7.82 ± 2.66
	*p*-value	0.83	0.06	0.30	0.70	0.01

I. Satisfaction with the ability to use pain relief methods in the course of childbirth. II. Satisfaction in having expectations and the birth plan well aligned to the actual course of labor. III. Satisfaction from interactions with medical personnel during childbirth. IV. Satisfaction from the possibility of using facilities during childbirth. V. Satisfaction with the overall birth experience. M—median, SD—standard deviation.

## Data Availability

Data available in a publicly accessible repository.
